# Accelerating cine DENSE using a zonal excitation

**DOI:** 10.1186/1532-429X-18-S1-O50

**Published:** 2016-01-27

**Authors:** Andrew D Scott, Upasana Tayal, Sonia Nielles-Vallespin, Pedro Ferreira, Xiaodong Zhong, Frederick H Epstein, Sanjay K Prasad, David Firmin

**Affiliations:** 1 grid.439338.6Cardiovascular Biomedical Research Unit, The Royal Brompton Hospital, London, UK; 2 grid.7445.20000000121138111National Heart and Lung Institute, Imperial College London, London, UK; 3 grid.279885.90000000122934638National Heart Lung and Blood Institute, National Institutes of Health, Bethesda, MD USA; 4 MR R&D Collaborations, Siemens Healthcare, Atlanta, GA USA; 5 grid.27755.32000000009136933XDepartment of Bioengineering, University of Virginia, Charlottesville, VA USA

## Background

Displacement encoding with stimulated echoes (DENSE) is an accurate and reproducible [1, 2] technique for measuring myocardial strain throughout the cardiac cycle. The strain maps provided could be valuable in dilated cardiomyopathy (DCM) patients where strain has a prognostic value [3]. However, DENSE acquisitions typically require long breath holds which are difficult for DCM patients. In this work we accelerate DENSE acquisitions by selectively exciting a volume of tissue around the heart. This allows fewer spiral interleaves to be acquired without aliasing and, therefore a shorter breath hold.

## Methods

**Figure 1:**
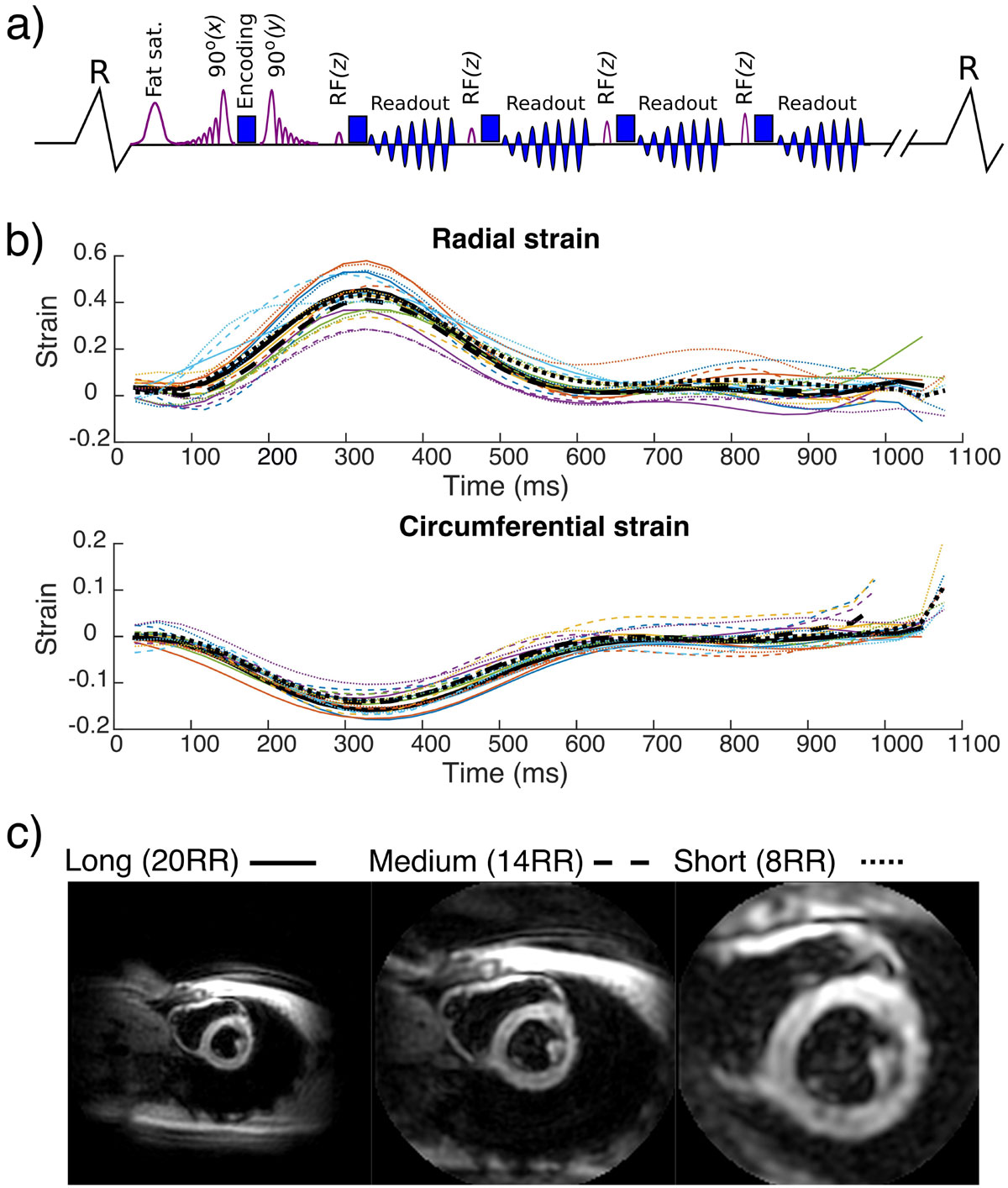
**Sequence schematic for the cine spiral DENSE sequence with zonal excitation (a)**. Example strain curves (b) and magnitude images (c) acquired in one example subject. Data from the long, medium and short acquisitions are shown as solid, dashed and dotted lines respectively. The coloured lines show the mean strain values from 6 equal angle segments and the black lines show the global short axis mean values. There is a good agreement between all three acquisitions.

A cine spiral DENSE sequence [4] was modified to selectively excite a reduced field of view. A slice selective gradient for the first and second RF pulses was added on the read and phase axes respectively (Figure 1). An improved excitation profile without increasing TE was achieved by making the 1st pulse asymmetric (peak at 81% of duration) and the 2nd pulse a time-reversed copy of the first.

In-vivo 2D cine DENSE was performed in 8 normal subjects (Siemens Skyra) in a mid short-axis slice. Images were acquired with variable flip angle (20° max), TE = 1 ms, fat suppression, 3.5 × 3.5 × 8 mm^3^ spatial resolution, 128^2^ matrix, 30 ms temporal resolution, 2 spirals/frame (6 ms/spirals), 2 direction encoding (+reference) at 0.06 cycles/mm, CSPAMM and through-plane dephasing artifact suppression. Long, medium and short acquisitions were performed with square field of view/breath hold duration of 360 mm/20RR-intervals (RR), 224 mm/14RR and 120 mm/8RR. The long acquisition used a similar field of view to previous work performed without the zonal excitation and was used as a reference [4]. Images were processed using the DENSE analysis tool from the University of Virginia [5].

## Results

The figure shows example magnitude images (c) and strain curves (b) acquired using long, medium and short acquisitions. There is good agreement between the strain curves from all three acquisitions. Global peak strain, time to peak strain and the differences between acquisitions (long as reference, expressed as bias and root mean square error) are provided in the table for radial and circumferential strain. The differences between the three acquisitions are small. Medium and short breath hold acquisitions appear to be equally accurate.

## Conclusions

Spiral cine DENSE imaging can be accelerated by up to a factor of 2.5 by selectively exciting and imaging a small field of view around the heart. The associated loss of signal to noise ratio is partly compensated for by imaging at 3T. This improvement allows 2D acquisitions to be performed in a short breath hold (~8s) which DCM patients could sustain. In future the same approach may be used to accelerate 3D spiral cine DENSE acquisitions.

**Table 1:** Global strain results

Acquisition	Peak strain	Bias in peak strain	Time to peak (ms)	Bias in time to peak (ms)	RMSE
Radial					
Long (reference)	0.40 ± 0.09	-	293 ± 32	-	-
Medium	0.39 ± 0.07	0.00 ± 0.07	312 ± 15	-19 ± 26	0.05
Short	0.41 ± 0.12	-0.01 ± 0.05	305 ± 25	-11 ± 30	0.05
Circumferential					
Long (reference)	-0.17 ± 0.01	-	320 ± 20	-	-
Medium	-0.16 ± 0.02	-0.01 ± 0.01	312 ± 15	8 ± 13	0.005
Short	-0.16 ± 0.02	-0.01 ± 0.01	320 ± 25	0 ± 15	0.003

RMSE: Root mean square error between the global strain curves.
